# Body mass index and body weight change during adjuvant chemotherapy in colon cancer patients: results from the AVANT trial

**DOI:** 10.1038/s41598-020-76643-9

**Published:** 2020-11-10

**Authors:** Dae-Won Lee, Sooyoung Cho, Aesun Shin, Sae-Won Han, Tae-You Kim

**Affiliations:** 1 Department of Internal Medicine, Seoul National University Hospital, 101 Daehang-Ro, Jongno-Gu, Seoul, 03080 Republic of Korea; 2 Department of Preventive Medicine, Seoul National University College of Medicine, 103 Daehang-Ro, Jongno-Gu, Seoul, 03080 Republic of Korea; 3 Cancer Research Institute, Seoul National University College of Medicine, Seoul, Republic of Korea; 4 Department of Molecular Medicine and Biopharmaceutical Sciences, Graduate School of Convergence Science and Technology, Seoul National University, Seoul, Republic of Korea

**Keywords:** Gastrointestinal cancer, Colorectal cancer

## Abstract

While obesity increases colorectal cancer incidence, there are inconsistent results in the prognostic role of obesity or body weight change on survival. This study investigated the prognostic impact of body weight and weight change in stage III or high risk stage II colon cancer patients. We used data from patients enrolled in the phase III AVANT trial. The AVANT trial investigated the efficacy of adding bevacizumab to standard adjuvant chemotherapy (FOFOX or XELOX). Weight change during the first 6 months of adjuvant chemotherapy was measured. Cox proportional hazard model was used to assess the prognostic influence of body weight and weight change. Among 3451 intention-to-treat population, body weight and weight change was measured in 3449 (99.9%) and 2455 (71.1%) patients, respectively. Among 2455 patients, 651 (26.5%) had weight gain over 5 kg and 179 (7.3%) had weight loss over 5 kg. Weight gain was more frequently observed in Asian and male. Neither baseline BMI nor weight change affected recurrence or survival in the Cox proportional hazard model.

## Introduction

Development of colorectal cancer is a heterogeneous process affected by hereditary factor, diet, and gut microbiome^1,2^. Obesity may increase colorectal cancer incidence through alternating adipocytokine levels and inducing insulin resistance^3,4^. It is suggested that obesity is associated with 30–70% increase of colorectal cancer incidence in men, although the association between obesity and colorectal cancer incidence is less consistent in women^3^. Approximately 35% of United States adults are obese and obesity prevalence is increasing in many developing countries that adopt Western lifestyle^5,6^. These findings may partially attribute to the increase of colorectal cancer incidence in developing countries^7,8^.

Obesity increases colorectal cancer incidence, but the prognostic role of obesity in patients diagnosed with colorectal cancer remains less conclusive. Although obesity did not impact cancer outcome in several studies^9,10^, most studies revealed negative impact of obesity on cancer survival^11–14^. Obesity may also have negative prognostic role in colorectal cancer patients treated with adjuvant chemotherapy^12,13^. However, the impact of obesity on cancer outcome is inconsistent according to sex. In a study by Meyerhardt et al., which 3759 patients were included, obesity was associated with poor survival in women but not in men^11^. In contrast, negative prognostic role of obesity was shown in men but not in women in the Adjuvant Colon Cancer Endpoints database, which 25,291 colon cancer patients were analyzed^12^.

Body weight change after surgery and during the adjuvant chemotherapy period is frequently observed^15^. However, only few studies have evaluated the association between body weight change and colorectal cancer outcome. Initially, Meyerhardt et al. reported that body weight change did not impact cancer recurrence or death^9^. However, body weight loss was a negative prognostic factor in following studies^16,17^. Using a single center retrospective data, we have previously reported that body weight gain during adjuvant chemotherapy period may have detrimental effect in overweight or obese patients^10^. This study was planned to validate our previous finding in a prospective clinical trial.

To date, the prognostic role of body weight and body weight change during adjuvant chemotherapy period remains inconclusive. We examined the prognostic role of body weight and weight change in patients who participated in a phase III AVANT trial. AVANT trial investigated adding bevacizumab with either 5-fluorouracil/leucovorin/oxaliplatin (FOLFOX) or capecitabine/oxaliplatin (XELOX) in curatively resected stage III or high-risk stage II colon cancer patients^18^.

## Methods

### Study population

Patients enrolled in the AVANT trial was included (ClinicalTrial.gov Identifier: NCT00112918)^18^. Study designs, key inclusion criteria, and exclusion criteria have previously been described in detail^18^. Histologically confirmed stage III or high-risk stage II colon cancer were included. The AVANT trial was a 3-arm study comparing FOLFOX4-bevacizumab versus FOLFOX4 or XELOX-bevacizumab versus FOLFOX4. Patients were 1:1:1 randomly assigned to receive either FOLFOX 4, FOLFOX4-bevacizumab, or XELOX-bevacizumab. From Dec 2004 to June 2007, 3,451 patients were randomized on the treatment arm (1151 received FOLFOX 4, 1155 received bevacizumab-FOLFOX4, and 1145 received bevacizumab-XELOX). Protocol was approved by institutional review boards or ethics review committees at each participating sites and was conducted in accordance with the declaration of Helsinki (3451 patients from 330 centers in 34 countries worldwide were randomly assigned). In our institute, the study protocol was reviewed and approved by the institutional review board of Seoul National University Hospital, Seoul, Korea [H-0412-139-011]. Written informed consent was provided by all participants before study enrollment. Access to the database by authors were approved by the sponsor Roche from “ClinicalStudyDataRequest.com”.

### Body mass index (BMI) and body weight change

Body mass index (BMI) was calculated by person's weight in kilograms (kg) divided by the height in meters squared (kg/m^2^). Body weight measured on study enrollment was defined as baseline body weight. We categorized BMI as underweight (less than 18.5 kg/m^2^), normal (18.5–24.9 kg/m^2^), overweight (25.0–29.9 kg/m^2^), and obese (≥ 30 kg/m^2^). Body weight measured 6 months after chemotherapy initiation was used as post-treatment weight. Body weight change during adjuvant chemotherapy period was measured by comparing post-adjuvant chemotherapy weight with baseline weight.

### Study end points

The primary objective was to investigate the impact of BMI and body weight change on disease-free survival (DFS). DFS was calculated from the date of randomization to recurrence, new occurrence of colorectal cancer, or death from any cause. Data from patients who were free of events were censored at the last date at which they were known to be disease-free. Secondary objective was to investigate the impact of BMI and body weight change on overall survival (OS). OS was calculated from the time of randomization to death from any cause. Patients who were free of events were censored at the known date of confirmed alive. Tumor assessment was pre-scheduled every 6 months after randomization until year 4, then annually thereafter.

### Statistical analysis

We compared baseline characteristics including age at entry, sex, disease stage, ECOG performance scale, chemotherapy regimen, ethnic origin, and smoking history by BMI groups using Chi square test and Fisher’s exact test. Cox proportional hazard model was used to estimate hazard ratios (HRs) and its corresponding 95% confidence intervals (CIs) of BMI and weight change for DFS and OS. We identified the best fitting model using likelihood ratio test and Akaike's information criterion^19,20^. Disease stage, ECOG performance scale and chemotherapy regimen were selected in the final model. Tests of significance were two-sided, with alpha level of 0.05. Statistical analyses were performed using R version 3.4 (R Foundation for Statistical Computing, Vienna, Austria).

## Results

### Baseline characteristics and survival outcome according to baseline BMI

Of the total of 3451 intention-to-treat population, baseline BMI was measured in 3449 patients. Patient characteristics according to baseline BMI are described in Table 1. According to the WHO definition, 93 patients (2.7%) were underweight (BMI < 18.5 kg/m^2^), 1643 (47.6%) had normal weight (18.5–24.9 kg/m^2^), 1239 (35.9%) had overweight (25–29.9 kg/m^2^), and 474 (13.7%) were obese (≥ 30 kg/m^2^). Underweight and normal weight patients were more likely to be younger and had higher proportion of female, never smoker, and Asian population compared to overweight and obese patients. ECOG performance scale, chemotherapy regimen, and disease stage was similar regardless of BMI groups (Table 1).

**Table 1:** Baseline characteristics according to baseline BMI.

	Total	Body Mass Index (kg/m^2^)				
		Underweight < 18.5	Normal 18.5–24.9	Overweight 25–29.9	Obese ≥ 30	
Total	3449	93 (2.7%)	1643 (47.6%)	1239 (35.9%)	474 (13.7%)	
**Age**						< 0.001
< 65 years	2456 (71.2%)	79 (84.9%)	1231 (74.9%)	816 (65.9%)	330 (69.6%)	
≥ 65 years	993 (28.8%)	14 (15.1%)	412 (25.1%)	423 (34.1%)	144 (30.4%)	
**Sex**						< 0.001
Male	1868 (54.2%)	25 (26.9%)	803 (48.9%)	768 (62.0%)	272 (57.4%)	
Female	1581 (45.8%)	68 (73.1%)	840 (51.1%)	471 (38.0%)	202 (42.6%)	
**Disease stage**						0.35
Stage II (high-risk)	577 (16.7%)	23 (24.7%)	286 (17.4%)	195 (15.7%)	73 (15.4%)	
Stage III, N1	1750 (50.7%)	42 (45.2%)	819 (49.8%)	641 (51.7%)	248 (52.3%)	
Stage III, N2	1122 (32.5%)	28 (30.1%)	538 (32.7%)	403 (32.5%)	153 (32.3%)	
**ECOG performance scale**						0.35
0	2959 (85.8%)	76 (81.7%)	1418 (86.3%)	1068 (86.2%)	397 (83.8%)	
1	487 (14.1%)	17 (18.3%)	224 (13.6%)	170 (13.7%)	76 (16.0%)	
(Missing)	3 (0.1%)	0 (0.0%)	1 (0.1%)	1 (0.1%)	1 (0.2%)	
**Chemotherapy regimen**						0.78
FOLFOX4	1150 (33.3%)	30 (32.3%)	554 (33.7%)	417 (33.7%)	149 (31.4%)	
Bevacizumab-FOLFOX4	1154 (33.5%)	27 (29.0%)	548 (33.4%)	408 (32.9%)	171 (36.1%)	
Bevacizumab-XELOX	1145 (33.2%))	36 (38.7%)	541 (32.9%)	414 (33.4%)	154 (32.5%)	
**Ethnic origin**						< 0.001*
Asian	434 (12.6%)	25 (26.9%)	319 (19.4%)	82 (6.6%)	8 (1.7%)	
White	2893 (83.9%)	61 (65.6%)	1279 (77.8%)	1105 (89.2%)	448 (94.5%)	
Other	122 (3.5%)	7 (7.5%)	45 (2.7%)	52 (4.2%)	18 (3.8%)	
**Smoking history**						< 0.001
Never smoked	1471 (42.7%)	43 (46.2%)	714 (43.5%)	522 (42.1%)	192 (40.5%)	
Past smoker	778 (22.6%)	18 (19.4%)	329 (20.0%)	299 (24.1%)	132 (27.8%)	
Current smoker	241 (7.0%)	9 (9.7%)	141 (8.6%)	66 (5.3%)	25 (5.3%)	
Data missing	959 (27.8%)	23 (24.7%)	459 (27.9%)	352 (28.4%)	125 (26.4%)	

*Fisher's exact test.

Among 3449 patients, 854 (24.8%) patients experienced cancer recurrence, and 459 (13.3%) patients died. The relation between baseline characteristics and DFS is shown in Table 2. The mean follow-up period was 37.8 months. Older age, stage III disease, and poor ECOG performance status was associated with worse DFS. However, baseline BMI status did not affect DFS (Table 2) or OS (Supplementary Table 1). Subgroup analysis according to ethnic was done and baseline BMI did not affect DFS nor OS in each ethnic group.

**Table 2:** Univariate analysis for disease-free survival.

	Total	∑F/U months	DFS event	Crude HR
**Age**				
< 65 years	2,456	93,666.6	580	Ref
≥ 65 years	993	36,896.7	274	1.20 (1.04–1.39)
**Sex**				
Male	1868	70,752.6	479	Ref
Female	1581	59,810.7	375	0.93 (0.82–1.07)
**Disease stage**				
Stage II (high-risk)	577	24,622.8	83	Ref
Stage III N1	1750	67,418.8	367	1.55 (1.22–1.97)
Stage III N2	1122	38,521.7	404	2.99 (2.36–3.79)
**ECOG performance scale**				
0	2959	113,016.6	695	Ref
1	487	17,398.3	159	1.48 (1.25–1.76)
(Missing)	3			
**Chemotherapy regimen**				
FOLFOX4	1150	43,882.0	263	Ref
FOLFOX4 + Bevacizumab	1154	43,790.2	309	1.17 (0.99–1.38)
XELOX + Bevacizumab	1145	42,891.1	282	1.09 (0.92–1.29)
**Ethnic origin**				
White	2893	110,598.1	731	Ref
Asian	434	15,815.8	94	0.87 (0.70–1.08)
Other	122	4149.3	29	1.04 (0.72–1.51)
**Smoking history at baseline**				
Never smoked	1471	52,766.8	359	Ref
Past smoker	778	28,253.3	181	0.94 (0.79–1.12)
Current smoker	241	8486.6	60	1.05 (0.80–1.37)
Data missing	959			
**Baseline BMI (kg/m**^**2**^**)**				
< 18.5: underweight	93	3608.9	19	0.78 (0.49–1.23)
18.5–24.9: normal	1643	61,241.4	419	Ref
25.0–29.9: overweight	1239	47,656.4	309	0.96 (0.83–1.11)
30.0–34.9: moderately obese	381	14,722.2	86	0.85 (0.68–1.08)
≥ 35: severely obese	93	3334.4	21	0.93 (0.60–1.44)

### Body weight change and colon cancer outcome

Body weight change during the first 6 months of adjuvant chemotherapy was measured. Among 3449 patients with baseline BMI, 2455 (71.1%) patients had data of body weight measured 6 months after the initiation of adjuvant chemotherapy. To identify the effect of body weight change during chemotherapy treatment, 2284 patients who received at least 50% of scheduled chemotherapy was included in the analysis of body weight change. Three different parameters were analyzed; absolute body weight change (≤ − 10 kg, − 9.9 to − 5 kg, − 4.9 to 4.9 kg, 5 to 9.9 kg, ≥ 10 kg), relative body weight change (≤ − 10%, − 9.9 to − 5%, − 4.9 to 4.9%, 5 to 9.9%, ≥ 10%), and BMI change (≤ − 2 kg/m^2^, − 1.9 to 1.9 kg/m^2^, ≥ 2 kg/m^2^). Detailed body weight change according to each parameter are shown in Table 3. Body weight change was frequent during the course of adjuvant chemotherapy. Among 2455 patients, 179 (7.3%) had weight loss over 5 kg and 651 (26.5%) had weight gain over 5 kg. The pattern of body weight change was different according to patient’s ethnicity and sex. Asian patients had higher proportion of weight gain over 5 kg (26.6% vs. 22.9%) and lower proportion of weight loss over 5 kg (1.8% vs. 6.7%) compared to White. In addition, weight gain over 5 kg was more frequent in male compared to women (27.2% vs. 20.3%, *p* = 0.004). We next evaluated the prognostic value of body weight change. Body weight change in all three parameter was not associated with DFS or OS (Table 3, Supplementary Table 2). Body weight change still did not impact DFS after adjusting for disease stage, ECOG performance status, and chemotherapy regimen. Subgroup analysis was performed to identify the impact of body weight change according to baseline BMI status, sex, and ethnic origin. However, there was no correlation between body weight change and DFS or OS in any subgroups. Detailed data of body weight change and DFS according to sex and age are shown in Supplementary Tables 3 and 4, respectively.

**Table 3:** Body weight change and disease-free survival.

	Total (*N* = 2455)	∑F/U months	DFS event	Crude HR	Adjusted HR
**Weight change (kg)**					
≤ − 10	31 (1.3%)	1195.2	10	1.43 (0.76–2.67)	1.19 (0.63–2.23)
− 9.9 to − 5	148 (6.0%)	6218.9	30	0.81 (0.56–1.18)	0.81 (0.56–1.17)
− 4.9 to 4.9	1625 (67.4%)	65,781.4	390	Ref	Ref
5 to 9.9	520 (21.2%)	21,139.1	128	1.03 (0.84–1.26)	1.07 (0.88–1.31)
≥ 10	131 (5.3%)	5485.2	27	0.84 (0.57–1.24)	0.85 (0.58–1.26)
**Weight change (%)**					
≤ − 10	77 (3.1%)	3062.3	21	1.16 (0.74–1.80)	1.06 (0.68–1.65)
− 9.9 to − 5	175 (7.1%)	7303.6	39	0.91 (0.65–1.27)	0.92 (0.66–1.28)
− 4.9 to 4.9	1320 (53.8%)	53,287.7	316	Ref	Ref
5 to 9.9	543 (22.1%)	22,125.8	131	1.01 (0.82–1.24)	1.03 (0.84–1.26)
≥ 10	340 (13.8%)	14,040.5	78	0.94 (0.74–1.21)	0.97 (0.76–1.25)
**Change in body mass index (kg per m**^**2**^**)**					
≤ − 2	139 (5.7%)	5678.1	34	1.02 (0.72–1.44)	0.97 (0.68–1.37)
− 1.9 to 1.9	1801 (73.3%)	73,019.0	431	Ref	Ref
≥ 2	515 (21.0%)	21,122.6	120	0.97 (0.79–1.19)	1.00 (0.82–1.23)

Adjusted for disease stage, ECOG performance scale, chemotherapy regimen.

## Discussion

Although obesity increases colon cancer incidence, prognostic role of obesity and body weight change remains inconclusive. This study investigated the prognostic effect of baseline BMI and body weight change in stage III and high-risk II colon cancer patients using the data of the AVANT trial. We selected AVANT trial to prove the hypothesis as patients included in this study received either FOLFOX or XELOX which is the standard treatment of choice in stage III colon cancer after complete resection of the tumor^21,22^. This study results shows baseline BMI or body weight change does not impact colon cancer recurrence or survival.

Many studies investigated the prognostic role of obesity in patients diagnosed with colorectal cancer^9–14^. Meyerhardt et al. reported that obesity (BMI ≥ 30 kg/m^2^) was associated with poor survival in women (hazard ratio [HR] of 1.34 for mortality) but not in men in 3759 high-risk stage II and stage III colon cancer patients^11^. However, the negative impact of obesity in women was not shown in the ACCENT database which included 25,291 colon cancer patients^12^. In the multivariate analysis, obesity was associated with poor survival in men but not in women^12^. Other studies showed negative impact of obesity on survival but sex difference was not shown or data on sex was not provided^13,14^. Evidences show that the risk of obesity on colorectal cancer development may be sex specific^3^. There is a hypothesis that insulin and insulin-like growth factor axis in colorectal neoplasia is different between sex, and this may partially explain the different role of obesity among sex^23^. Moreover, the risk of obesity on colorectal cancer risk is different among menopausal status^24^. Obesity was associated with colorectal cancer risk in premenopausal but not in postmenopausal women. Sex and hormone status may have contributed to the conflicting results of previous studies. However, in the present study, baseline BMI did not affect recurrence or survival in any subgroup of patients. Obesity was not associated with cancer outcome regardless of sex and age. We believe future larger cohort studies are needed to confirm these findings.

Life style modification and weight change are very common in patients diagnosed with cancer^25^. Physical activity reduces the risk of cancer recurrence and overall mortality in colorectal cancer patients^26^. In the present study, body weight gain was more frequent during adjuvant chemotherapy period compared to body weight loss. Twenty-five percent of patients had weight gain over 5 kg during adjuvant chemotherapy and 7.3 percent had weight loss over 5 kg. Weight gain was more frequently observed in Asian and male population, respectively. While body weight change during adjuvant chemotherapy period is frequently observed, only few studies have investigated the prognostic role of body weight change on colorectal cancer outcome. Neither weight gain nor loss was associated with cancer recurrence or death in 1053 stage III colon cancer patients treated with adjuvant chemotherapy in a clinical trial (CALGB 89803)^9^. However, in a population-based longitudinal study of 1825 stage I to III colorectal cancer patients, weight loss over 5 kg was associated with increased mortality (HR of 1.63) while weight gain did not impact cancer outcome^27^. The negative prognostic impact of weight loss after diagnosis was also shown in 2781 stage I-III colorectal cancer patients from Kaiser Permanente Northern California population^17^. Loss of 10% or more body weight was associated with increase in colorectal cancer-specific mortality (HR of 3.20)^17^. In breast cancer patients, although controversial, many studies showed possible negative prognostic role of body weight gain after cancer diagnosis^28,29^. However, in colorectal cancer, only 1 study showed negative prognostic role of body gain after diagnosis. In a single institution retrospective study, body weight gain (5 kg or more) in overweight or obese patients was associated with poor outcome^10^. Among 522 stage III or high-risk stage II colorectal cancer patients treated with adjuvant FOLFOX in South Korea, body weight gain (≥ 5 kg) during adjuvant chemotherapy was associated with inferior disease-free survival (DFS) (HR of 2.04) in overweight and obese patients^10^. In the present study, body weight gain or loss was not associated with recurrence or survival. Weight change still did not impact cancer outcomes when stratified by ethnic, sex, age, or baseline BMI. Weight change during adjuvant chemotherapy may not affect recurrence or survival in colon cancer treated with curative surgery followed by adjuvant FOLFOX/XELOX chemotherapy.

The limitation of the present study is that we could not assess other measures associated with metabolic dysregulation including waist circumference, waist-hip ratio, physical activity, and metabolic syndrome. The purpose of this study was not pre-defined in the clinical trial, so these measures were not collected. As body fat composition and distribution is affected by sex, age, and ethnics, using BMI may have pitfalls in assessing the true extent of obesity^30^. These limitations may have affected inconsistent results of baseline BMI and body weight change on prognosis of cancer patients. Nevertheless, BMI is a simple measure of obesity which is readily available and commonly used. Future comprehensive analysis of obesity is needed to evaluate the association between metabolic dysregulation and cancer outcome. In addition, evaluating adipocytokines level such as leptin, resistin, and adiponectin will help physicians to understand metabolic status of a patient. Another limitation of this study is that the time between body weight measurements is relatively short (6 months). Long-term body weight change might have different effect on prognostic outcome and future study is warranted.

## Conclusion

This study shows that neither BMI nor weight change were associated with cancer recurrence or survival in patients enrolled in the AVANT trial.

## Supplementary information

Supplementary Tables.

## Data Availability

Any requested data and materials will be considered by the corresponding author.
