# Supplementary material for: Body mass index and body weight change during adjuvant chemotherapy in colon cancer patients: results from the AVANT trial
Source: Sci Rep. 2020 Nov 10;10:19467. doi: 10.1038/s41598-020-76643-9 (PMC7655869; doi:10.1038/s41598-020-76643-9)
Supplement: Supplementary file 1 — Supplementary Tables. [file 41598_2020_76643_MOESM1_ESM.docx]

**Body Mass Index and Body Weight Change During Adjuvant Chemotherapy in Colon Cancer Patients**

**: Results From the AVANT Trial**

Dae-Won Lee^1,*^, Sooyoung Cho^2,*^, Aesun Shin^2,3**^,

Sae-Won Han^1,3**^, and Tae-You Kim^1,3,4^

^1^Department of Internal Medicine, Seoul National University Hospital, Seoul, Korea;

^2^Department of Preventive Medicine, Seoul National University College of Medicine, Seoul, Korea;

^3^Cancer Research Institute, Seoul National University College of Medicine, Seoul, Korea;

^4^Department of Molecular Medicine & Biopharmaceutical Sciences, Graduate School of Convergence Science and Technology, Seoul National University, Seoul, Korea

*These authors contributed equally as first authors.

****Corresponding authors**

Aesun Shin, MD, PhD

Department of Preventive Medicine

Seoul National University College of Medicine

103 Daehang-Ro, Jongno-Gu

Seoul, 03080, Republic of Korea

Tel: 82-2-740-8331 Fax: 82-2-747-4830

E-mail: shinaesun@snu.ac.kr

Sae-Won Han, MD, PhD

Department of Internal Medicine

Seoul National University Hospital

101 Daehang-Ro, Jongno-Gu

Seoul, 03080, Republic of Korea

Tel: 82-2-2072-0242 Fax: 82-2-762-9662

E-mail: [saewon1@snu.ac.kr](mailto:saewon1@snu.ac.kr)

**Supplement Table 1. Univariate analysis for overall-survival**

|  | **Total** | **Follow-up**  **period (months)** | **Death** | **Crude HR** | **95% CI** | |
| --- | --- | --- | --- | --- | --- | --- |
| Age |  |  |  |  |  |  |
| < 65 years | 2,456 | 109,539.3 | 301 | ref. |  |  |
| ≥ 65 years | 993 | 43,265.7 | 158 | 1.33 | (1.10 | -1.62) |
| Sex |  |  |  |  |  |  |
| Male | 1,868 | 82,671.5 | 259 | ref. |  |  |
| Female | 1,581 | 70,133.4 | 200 | 0.91 | (0.76 | -1.09) |
| Disease Stage |  |  |  |  |  |  |
| Stage II (high-risk) | 577 | 28,097.4 | 46 | ref. |  |  |
| Stage IIIN1 | 1,750 | 78,043.4 | 172 | 1.37 | (0.99 | -1.89) |
| Stage IIIN2 | 1,122 | 46,664.2 | 241 | 3.24 | (2.36 | -4.44) |
| ECOG performance scale |  |  |  |  |  |  |
| 0 | 2,959 | 131,659.8 | 357 | ref. |  |  |
| 1 | 487 | 20,984.4 | 102 | 1.80 | (1.45 | -2.25) |
| (Missing) | 3 |  | 0 |  |  |  |
| Chemotherapy regimen |  |  |  |  |  |  |
| FOLFOX4 | 1,150 | 50,920.1 | 134 | ref. |  |  |
| FOLFOX4 + Bevacizumab | 1,154 | 51,195.4 | 168 | 1.25 | (1.00 | -1.57) |
| XELOX + Bevacizumab | 1,145 | 50,689.5 | 157 | 1.18 | (0.94 | -1.48) |
| Ethnic origin |  |  |  |  |  |  |
| White | 2,893 | 129,435.7 | 392 | ref. |  |  |
| Asian | 434 | 18,211.2 | 50 | 0.91 | (0.68 | -1.22) |
| Other | 122 | 5,158.1 | 17 | 1.10 | (0.67 | -1.78) |
| Smoking history at baseline |  |  |  |  |  |  |
| Never smoked | 1,471 | 61,781.7 | 183 | ref. |  |  |
| Past smoker | 778 | 32,745.4 | 99 | 1.02 | (0.80 | -1.30) |
| Current smoker | 241 | 10,205.3 | 35 | 1.16 | (0.81 | -1.66) |
| (Missing) | 959 |  | 142 |  |  |  |
| Body mass index(kg per m^2^) |  |  |  |  |  |  |
| < 18.5: Underweight | 93 | 4,138.3 | 8 | 0.60 | (0.29 | -1.20) |
| 18.5 - 24.9: Normal | 1,643 | 72,307.8 | 234 | ref. |  |  |
| 25.0 - 29.9: Overweight | 1,239 | 55,577.0 | 148 | 0.82 | (0.67 | -1.01) |
| ≥ 30: Obese | 474 | 20,781.9 | 69 | 1.02 | (0.78 | -1.34) |

**Supplement Table 2. Body weight change and overall survival**

|  | **Total** | **Follow-up period**  **(months)** | **Death** | **Crude HR** | **95% CI** | | **Adjusted HR^a^** | **95% CI** | | **Adjusted HR^b^** | **95% CI** | |
| --- | --- | --- | --- | --- | --- | --- | --- | --- | --- | --- | --- | --- |
| Weight change (kg) |  |  |  |  |  |  |  |  |  |  |  |  |
| ≤ -10 | 31 | 1,412.1 | 4 | 1.18 | (0.44 | -3.17) | 0.96 | (0.36 | -2.59) | 0.91 | (0.33 | -2.47) |
| -9 to -5 (-10<, ≤-5) | 148 | 6,915.6 | 18 | 1.07 | (0.66 | -1.74) | 1.08 | (0.67 | -1.76) | 1.05 | (0.64 | -1.71) |
| -4 to 4 (-5<, <5) | 1,625 | 75,332.1 | 182 | ref. |  |  | ref. |  |  | ref. |  |  |
| 5 to 9 | 520 | 24,410.4 | 70 | 1.18 | (0.90 | -1.56) | 1.22 | (0.93 | -1.61) | 1.22 | (0.92 | -1.62) |
| ≥ 10 | 131 | 6,251.1 | 16 | 1.05 | (0.63 | -1.75) | 1.02 | (0.61 | -1.71) | 1.05 | (0.62 | -1.76) |
| Weight change (%) |  |  |  |  |  |  |  |  |  |  |  |  |
| ≤ -10 | 77 | 3,531.8 | 10 | 1.16 | (0.61 | -2.21) | 1.04 | (0.55 | -1.98) | 1.01 | (0.53 | -1.93) |
| -9 to -5 (-10<, ≤-5) | 175 | 8,167.4 | 23 | 1.15 | (0.74 | -1.79) | 1.19 | (0.77 | -1.84) | 1.21 | (0.78 | -1.89) |
| -4 to 4 (-5<, <5) | 1,320 | 61,141.3 | 149 | ref. |  |  | ref. |  |  | ref. |  |  |
| 5 to 9 | 543 | 25,274.9 | 65 | 1.06 | (0.79 | -1.41) | 1.10 | (0.82 | -1.47) | 1.11 | (0.83 | -1.50) |
| ≥ 10 | 340 | 16,205.9 | 43 | 1.08 | (0.77 | -1.52) | 1.07 | (0.76 | -1.51) | 1.11 | (0.78 | -1.57) |
| Change in body mass index(kg per m2) |  |  |  |  |  |  |  |  |  |  |  |  |
| ≤ -2 | 139 | 6,413.4 | 19 | 1.21 | (0.76 | -1.94) | 1.14 | (0.71 | -1.82) | 1.11 | (0.68 | -1.78) |
| -1 to 1 (-2<, <2) | 1,801 | 83,623.0 | 204 | ref. |  |  | ref. |  |  | ref. |  |  |
| ≥ 2 | 515 | 24,284.9 | 67 | 1.13 | (0.85 | -1.49) | 1.14 | (0.86 | -1.50) | 1.16 | (0.87 | -1.53) |

^a^ Adjusted for disease stage ( stage II [high-risk], stage IIIN1, stage IIIN2), ECOG performance scale (0,1, missing), chemotherapy regimen (FOLFOX4, FOLFOX4 + Bevacizumab, XELOX + Bevacizumab); ^b^ Adjusted for age group (< 65, ≥65 years), sex, disease stage ( stage II [high-risk], stage IIIN1, stage IIIN2), ECOG performance scale (0,1, missing), chemotherapy regimen (FOLFOX4, FOLFOX4 + Bevacizumab, XELOX + Bevacizumab), body mass index at basline (kg per m2; < 18.5 [underweight], 18.5 - 24.9 [normal), 25.0 - 29.9 [overweight], ≥ 30 [obese]), smoking history (nerver smoked, past smoker, current smoker, missing)

| **Men** | | | |  | **Women** | | | |  |
| --- | --- | --- | --- | --- | --- | --- | --- | --- | --- |
|  | **N** | **Follow-up period (months)** | **Case** | **Adjusted HR** |  | **N** | **Follow-up period (months)** | **Case** | **Adjusted HR** |
| Weight change, kg | |  |  |  |  |  |  |  |  |
| ≤-10 | 20 | 713.2 | 8 | 1.64 (0.81-3.33) |  | 11 | 482.0 | 2 | 0.57 (0.14-2.31) |
| -9.9 to -5 | 73 | 3,066.0 | 13 | 0.67 (0.38-1.18) |  | 75 | 3,153.0 | 17 | 0.94 (0.57-1.56) |
| -4.9 to 4.9 | 864 | 84,897.2 | 213 | ref. |  | 761 | 30,884.2 | 177 | ref. |
| 5 to 9.9 | 315 | 12,724.5 | 84 | 1.16 (0.90-1.49) |  | 205 | 8,414.6 | 44 | 0.93 (0.67-1.30) |
| ≥10 | 84 | 3,509.4 | 20 | 0.95 (0.60-1.50) |  | 47 | 1,975.8 | 7 | 0.66 (0.31-1.41) |
| Weight change, % | |  |  |  |  |  |  |  |  |
| ≤-10 | 35 | 1,315.8 | 12 | 1.36 (0.76-2.45) |  | 42 | 1,746.4 | 9 | 0.82 (0.42-1.62) |
| -9.9 to -5 | 71 | 3,007.6 | 12 | 0.64 (0.35-1.14) |  | 104 | 4,296.0 | 27 | 1.16 (0.77-1.76) |
| -4.9 to 4.9 | 748 | 30,260.3 | 184 | ref. |  | 572 | 23,027.3 | 132 | ref. |
| 5 to 9.9 | 329 | 13,152.0 | 83 | 1.10 (0.85-1.43) |  | 214 | 8,973.8 | 48 | 0.94 (0.67-1.31) |
| ≥10 | 173 | 7,174.5 | 47 | 1.12 (0.81-1.54) |  | 167 | 6,866.0 | 31 | 0.83 (0.56-1.23) |
| BMI change, % | |  |  |  |  |  |  |  |  |
| ≤-2 | 62 | 2,489.1 | 15 | 0.92 (0.55-1.55) |  | 77 | 3,189.0 | 19 | 1.01 (0.63-1.63) |
| -1.9 to 1.9 | 1,009 | 40,750.0 | 249 | ref. |  | 792 | 32,269.0 | 182 | ref. |
| ≥2 | 285 | 11,671.1 | 74 | 1.09 (0.84-1.42) |  | 230 | 9,451.5 | 46 | 0.90 (0.65-1.24) |

Supplement table 3. Body weight change and Disease-free survival stratified by sex.

**Supplement table 4. Body weight change and Disease-free survival stratified by age.**

| **< 65 years** | | | |  | **≥ 65 years** | | | |  |
| --- | --- | --- | --- | --- | --- | --- | --- | --- | --- |
|  | **N** | **Follow-up period (months)** | **Case** | **Adjusted HR** |  | **N** | **Follow-up period (months)** | **Case** | **Adjusted HR** |
| Weight change, kg | |  |  |  |  |  |  |  |  |
| ≤-10 | 22 | 930.9 | 6 | 0.93 (0.41-2.09) |  | 9 | 264.3 | 4 | 1.98 (0.72-5.41) |
| -9.9 to -5 | 86 | 3,574.5 | 14 | 0.66 (0.39-1.14) |  | 62 | 2,644.5 | 16 | 0.91 (0.53-1.54) |
| -4.9 to 4.9 | 1,179 | 47,696.3 | 270 | ref. |  | 446 | 18,085.0 | 120 | ref. |
| 5 to 9.9 | 429 | 17,413.3 | 106 | 1.13 (0.90-1.41) |  | 91 | 3,725.8 | 22 | 0.94 (0.59-1.48) |
| ≥10 | 118 | 4,982.6 | 24 | 0.85 (0.56-1.30) |  | 13 | 502.7 | 3 | 1.11 (0.35-3.53) |
| Weight change, % | |  |  |  |  |  |  |  |  |
| ≤-10 | 35 | 1,315.8 | 12 | 1.36 (0.76-2.45) |  | 42 | 1,746.4 | 9 | 0.82 (0.42-1.62) |
| -9.9 to -5 | 71 | 3,007.6 | 12 | 0.64 (0.35-1.14) |  | 104 | 4,296.0 | 27 | 1.16 (0.77-1.76) |
| -4.9 to 4.9 | 748 | 30,260.3 | 184 | ref. |  | 572 | 23,027.3 | 132 | ref. |
| 5 to 9.9 | 329 | 13,152.0 | 83 | 1.10 (0.85-1.43) |  | 214 | 8,973.8 | 48 | 0.94 (0.67-1.31) |
| ≥10 | 173 | 7,174.5 | 47 | 1.12 (0.81-1.54) |  | 167 | 6,866.0 | 31 | 0.83 (0.56-1.23) |
| BMI change, % | |  |  |  |  |  |  |  |  |
| ≤-2 | 62 | 2,489.1 | 15 | 0.92 (0.55-1.55) |  | 77 | 3,189.0 | 19 | 1.01 (0.63-1.63) |
| -1.9 to 1.9 | 1,009 | 40,750.0 | 249 | ref. |  | 792 | 32,269.0 | 182 | ref. |
| ≥2 | 285 | 11,671.1 | 74 | 1.09 (0.84-1.42) |  | 230 | 9,451.5 | 46 | 0.90 (0.65-1.24) |
